# Information Theoretic-Based Interpretation of a Deep Neural Network Approach in Diagnosing Psychogenic Non-Epileptic Seizures

**DOI:** 10.3390/e20020043

**Published:** 2018-01-23

**Authors:** Sara Gasparini, Maurizio Campolo, Cosimo Ieracitano, Nadia Mammone, Edoardo Ferlazzo, Chiara Sueri, Giovanbattista Gaspare Tripodi, Umberto Aguglia, Francesco Carlo Morabito

**Affiliations:** 1 Department of Medical and Surgical Sciences, Magna Græcia University, 88100 Catanzaro, Italy; 2 Regional Epilepsy Centre, Bianchi-Melacrino-Morelli Hospital, 89124 Reggio Calabria, Italy; 3 Dipartimento di Ingegneria Civile, dell’Energia, dell’Ambiente e dei Materiali, DICEAM Department, University Mediterranea of Reggio Calabria, 89124 Reggio Calabria, Italy; 4 Istituto di Ricovero e Cura a Carattere Scientifico, IRCCS Centro Neurolesi Bonino-Pulejo, 98124 Messina, Italy

**Keywords:** psychogenic non-epileptic seizures, deep learning, stacked autoencoders, information theory, entropy

## Abstract

The use of a deep neural network scheme is proposed to help clinicians solve a difficult diagnosis problem in neurology. The proposed multilayer architecture includes a feature engineering step (from time-frequency transformation), a double compressing stage trained by unsupervised learning, and a classification stage trained by supervised learning. After fine-tuning, the deep network is able to discriminate well the class of patients from controls with around 90% sensitivity and specificity. This deep model gives better classification performance than some other standard discriminative learning algorithms. As in clinical problems there is a need for explaining decisions, an effort has been carried out to qualitatively justify the classification results. The main novelty of this paper is indeed to give an entropic interpretation of how the deep scheme works and reach the final decision.

## 1. Introduction

In recent years, Deep Learning (DL) has generated a resurgence of interest in machine learning and neural networks. DL is a technology that has provided impressive performance in such diverse application fields as robotics, visual object recognition, image classification, health, financial analysis and speech recognition [1,2,3,4]. In general, DL allows learning representative features of a problem hierarchically, from raw data [5,6]. Recently, researchers have exploited DL theory and developed deep models to aid the diagnosis, based only on electroencephalographic (EEG) data, of some neurological diseases, such as epilepsy, Alzheimer disease’s (AD), Mild Cognitive Impairment (MCI). EEG is a non-invasive, low-cost routinely tool, which records the electrical activity of the brain through a set of electrodes arranged in a cap according to a standard International System. Wulsin et al. [7] investigated epilepsy in EEG signals by using deep belief nets (DBNs); whereas, Mirowski et al. [8] combined Convolutional Neural Networks (CNNs) and wavelet coherence analysis for the prediction of epileptic seizures, achieving 71% sensitivity with zero false positives on 15 out of 21 patients of Freiburg dataset. Zhao et al. [9] proposed a stacked Restricted Boltzmann Machines (RBM) to discriminate EEG signals recorded in patients affected by AD from normal subjects, reporting 92% of accuracy. Morabito et al. [10] used a Stacked Auto-Encoder (SAE), based on unsupervised learning techniques, to distinguish patients with early-stage Creutzfeldt-Jakob disease (CJD) from other forms of rapidly progressive dementia (RPD), achieving an average 89% accuracy. Morabito et al. [11] also designed a CNN to classify EEG recordings of AD, MCI and Healthy Control (CNT) subjects. The proposed processor provided the following results in term of accuracy: 82% in AD vs. MCI vs. CNT classification, 85% in MCI vs. CNT classification, 85% in AD vs. CNT classification and 78% in AD vs. MCI classification.

Motivated by the promising results achieved by DL on clinical applications, the present paper introduces a novel deep architecture, which is able to differentiate patients affected by psychogenic non-epileptic seizures (PNES) from healthy control (CNT) subjects based on EEG recordings. PNES are short and sudden behavioral changes that resemble epileptic seizures, but do not exhibit EEG ictal patterns. In these subjects, there is no evidence of other possible somatic causes of the seizures, whereas there is strong evidence that such seizures are caused by psychogenic factors [12]. Many patients with PNES are erroneously diagnosed with epilepsy, and thus they may receive inappropriate treatment leading to inefficacy and relevant side effects [13,14]. This may be due to difficulties in getting a correct medical history of such patients and to the fact that interictal EEG is often normal in epilepsy patients. Thus, according to the International League against Epilepsy, PNES diagnosis is based on a stepwise approach, involving several clinical and neurophysiological examinations, to formulate a diagnosis with a growing level of certainty [15]. In particular, the diagnosis of definite PNES is based on the visual examination of seizures captured during video-EEG recording. PNES may occur spontaneously during long-term EEG recording or may be evoked by means of suggestion techniques. While the former is costly for the national health system, the latter is not free from ethical concerns [16]. The challenge for neurologists is to achieve an early and accurate diagnosis of PNES, based on clinical data and standard EEG. Hence, the availability of an alternative method to diagnose PNES from interictal EEGs, with no need to capture the ictal events, would be of great benefit for both physicians and patients.

In this paper, a SAE-DL architecture is proposed to learn latent features of the EEGs recorded from PNES and CNT, specifically from their time-frequency (TF) representation. To our best knowledge this is the first study on PNES based on DL. The use of machine learning scheme has been shown to be beneficial for helping clinicians in diagnosing neurological diseases. However, in this setting, there is a strong need to explain the machine decisions. For this reason, in this paper, the DL approach is complemented by an attempt to interpret the learned representation in terms of an information theoretic methodology. This approach represents the main novelty of the present work.

The paper is organized as follows: in Section 2, the available database is described, and the proposed processing methodologies are presented. In Section 3, the performance of the designed DL model are reported and a suitable measure of entropy is carried out giving hints on the way how DL works. In Section 4, some conclusions are drawn.

## 2. Materials and Methods

### 2.1. Subjects and Electrophysiological Recordings

The data analyzed in this work were collected from the Regional Epilepsy Centre, Great Metropolitan Hospital of Reggio Calabria, University of Catanzaro, Reggio Calabria, Italy. We evaluated six EEGs of six PNES patients (six F; age range 17–40 years, median 34.5) and of 10 EEG from 10 healthy control volunteers (CNT) (eight Females, 2 Males; age range 25–65 years, median 44). The diagnosis of PNES was based on the following criteria: (a) typical clinical events recorded by video-EEG examination, provoked by suggestion maneuvers; (b) EEG showing neither concomitant ictal activity, nor postictal slowing [12,13]. It is worth noting that all of the included EEG traces were recorded before inducing the seizures. None of enrolled subjects had been receiving any medication.

Every EEG was recorded by scalp electrodes placed at 19 standard locations according to the International 10–20 System (Fp1, Fp2, F3, F4, C3, C4, P3, P4, O1, O2, F7, F8, T3, T4, T5, T6, Fz, Cz and Pz) with referential montage using G2 (located between electrodes Fz and Cz) as reference. The EEGs have been acquired in the morning, in a comfortable resting state, with eyes closed. Subjects were asked to open and close their eyes 2–5 times during the exam to test the reactivity of background activity. The technician kept the subject alert to prevent drowsiness. The average recording length is 30 min. The EEG was high-pass filtered at 0.5 Hz, low-pass filtered at 70 Hz, plus 50 Hz notch filtered with slope of 12 dB/Oct, and then down-sampled to 256 Hz. The EEG frames showing evident artifacts were identified by visual inspection and cancelled. All patients and caregivers signed an informed consent form.

### 2.2. Time-Frequency Feature Extraction

Although visual inspection of EEG signals is often the gold standard for diagnosis, often it does not allow for the extraction all of the clinically relevant information embedded in the EEG: the EEG representation in the frequency or time-frequency domain can indeed yield some additional or alternative insights on the recording. In this work, the time-frequency representation is obtained by using the Continuous Wavelet Transform (CWT). It is generated by passing time-windows of the EEG signal (epochs) through a wavelet filter of finite predetermined length (the “mother” wavelet is the prototype function which is scaled and shifted to match the original time signal at different scales). The CWT is computed by multiplying the scaled and shifted versions of the mother wavelet by the EEG epoch under consideration and then integrating the product in time, as in Equation (1):

(1) $$\mathrm{CWT}\left(\alpha ,\tau \right)=\frac{1}{\sqrt{\alpha }}\int_{-T}^{T}eeg\left(t\right)\Psi^{*}\left(\frac{t-\tau }{\alpha }\right)dt$$

In (1), *α* is the scale factor, *τ* the time delay, and *Ψ* (•) is the selected mother wavelet. The length of the epoch is 2*T*. A high value of the CWT coefficient reflects a relevant spectral component of the signal at *α* and *τ*. The “Mexican hat” mother wavelet is here used:

(2) $$\Psi \left(t\right)=\frac{1}{\sigma^{3}\sqrt{2\pi }}\left(1-\frac{t^{2}}{\sigma^{2}}\right)e^{\frac{-t^{2}}{2\sigma^{2}}}$$

### 2.3. Deep Learning (DL) Approach

DL is a learning paradigm used to train multilayered neural networks acting as multiple processing layers. The successive layers gradually infer and extract, through learning from big data, a compressed representation of the input in terms of a set of features [17,18]. In conventional approaches, the same scheme is shallow; just one hidden layer is placed between the input coming from sensors and the output layer. In both cases the data are processed to solve a specific classification task. The network model here proposed includes a first representational stage which consists in transforming the EEG time vectors in a two-dimensional time-frequency map. Then some “engineered” features are extracted from the maps. Such features form the input to various stages of stacked auto-encoder. Each compressed version is the encoded representation of the input vector generated by means of a bottleneck encoder-decoder scheme. Each successive representation is a set of features extracted from the previous representation [19]. The learning of the autoencoders is unsupervised, i.e., it does not make use of the known label associated with the class. The output vector of the deepest hidden layer is used as input vector to a final classification stage, based on a standard neural network trained with supervised learning. Sometimes, an improvement of the performance of the whole deep network can be achieved by a supervised fine-tuning step. In this case, the procedure is affected from the gradient dilution problem that invariably limits in naïve implementations the quality of error back-propagation [18]. As DL schemes typically involve a huge number of degrees of freedom, specific cost functions are commonly defined to reach an optimal trade-off between the accuracy and the sparsity of the representation. The relative importance of the two parts is managed through a regularization coefficient [12,17,18].

### 2.4. DL-Based Processing System for EEG Classification

**Figure 1:**
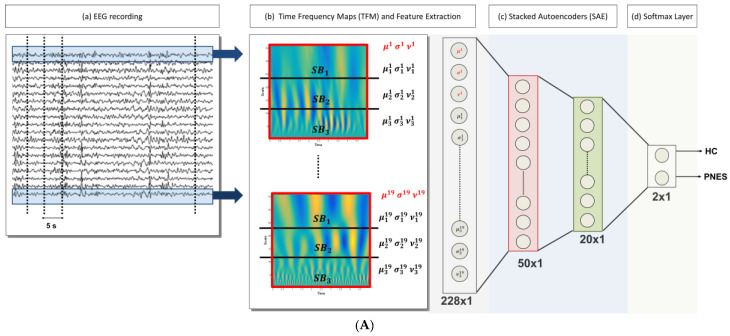

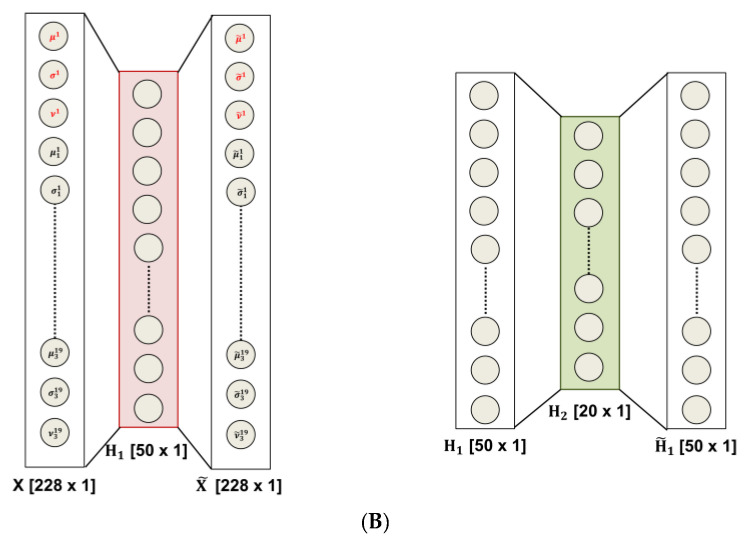
(**A**) The flowchart of the method: (a) The 19-channels electroencephalography (EEG) recording is partitioned into M = 20 non-overlapping epochs (of 5 s width), (b) given an epoch, the time frequency map (TFM) is estimated over each channel. The *i*-th TFM (*i* = 1, …, 19) is partitioned into three sub-bands ($SB_{j}$ *j* = 1, 2, 3); then, the mean (*µ*), standard deviation (*σ*), and skewness (*ν*) of the wavelet coefficients are evaluated for each *SB* and for the whole TFM. Once the TFMs are computed on the M = 20 epochs, a database of 20 × 12 × 19 data (#epochs × #features × #channels) is generated, (c) The vectors of 228 features are the input of a 2-stacked autoencoders (SAE) architecture. The last softmax layer performs the 2-way classification task (CNT-PNES); (**B**) The two Autoencodes (AE) implemented: the first AE compresses the 228 input features to 50 parameters (encoder stage) and then attempts to reconstruct the input (decoder stage); whereas, the second AE compresses the 50 features output of the first AE to 20 latent parameters. The compressed representations H_1_ (50 × 1) and H_2_ (20 × 1) (indicated in red and green, respectively) are used in the stacked autoencoders architecture.

**Figure 2:**
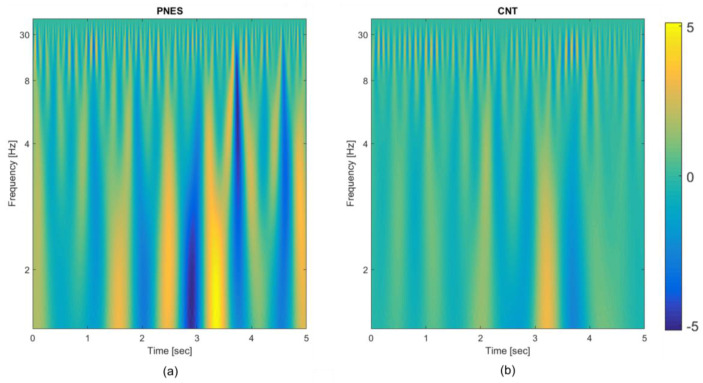
Time frequency representation of the psychogenic non-epileptic seizures (PNES) and healthy control (CNT). Each epoch of the 19-channels electroencephalography (EEG) is transformed in a time frequency map (TFM); then, the mean over the 19 channels, over the subjects and over the epochs is evaluated coming up with a single TFM per class. (**a**) TFM averaged over the 19 channels, the 20 epochs, and the six PNES subjects; (**b**) TFM averaged over 19 channels, the 20 epochs, and the 10 CNT subjects.

**Figure 3:**
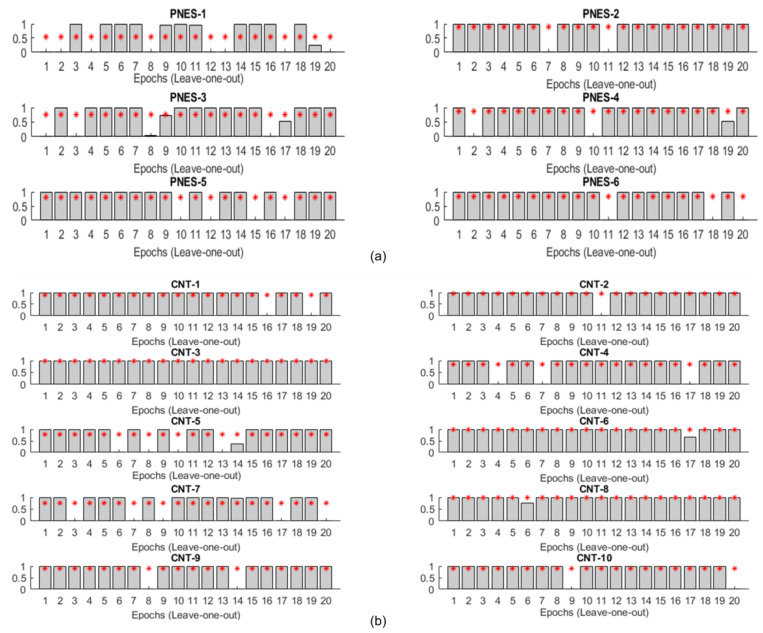
Softmax output representation of PNES (**a**) and CNT (**b**) for the 20 leave-one-out testing sessions carried out for every subject. Each bin represents the output estimated by the softmax layer ranged between 0 and 1 (1 correct classification; 0 misclassification). The red dotted line is the average output level of the network, evaluated over the 20 sessions.

In what follows, the proposed DL-based processing scheme of the EEG signals is synthetically described. Figure 1 pictorially represents the architecture of the processing system here designed. The available EEG database, which includes all of the subjects from both categories, i.e., PNES and CNT, was processed in multiple steps, as reported below:

(1) Artifact rejection: rejection of the artifacts through the visual inspection of each EEG recording; the EEG segments clearly affected by artefactual components are discarded, Figure 1A (a);
(2) EEG signal decomposition: the cleaned EEG recording is subdivided in non-overlapping *T* = 5 s epochs, Figure 1A (a);
(3) TF transformation: each EEG epoch is time-frequency transformed by using CWT, as in (1), by using a Mexican hat function as mother wavelet (2), Figure 1A (b); the use of CWT showed significant advantages on simple spectrograms probably because of the choice of the mother wavelet function, which is particularly suitable for EEG signals;
(4) Engineered feature extraction: partitioning of the CWT map into three parts (sub-bands maps) and estimation of the mean value (*µ*), the standard deviation (*σ*) and the skewness (*υ*) either of the three sub-bands maps and of the whole CWT map, Figure 1A (b); the widths of the three non-overlapping sub-bands have been selected by an optimization algorithm and do not exactly correspond to the brain rhythms [12]; the two higher bands in Figure 1A (b) roughly include delta and theta rhythms;
(5) Preparation of the feature vector: the resulting feature vector includes three features per electrode (*µ*, *σ*, and *υ* for each of the three sub-bands maps and the *µ*, *σ*, and *υ* of the whole CWT map); thus, the input vector of the autoencoders chain has a length of 12 (features) × 19 (electrodes) = 228 elements, Figure 1A (c);
(6) Data-driven feature compression: two stages of autoencoding are used as compressors giving 50 and 20 successively extracted data-driven features; at this level the features extracted from each channel are combined outputting an unsupervised learned vector that mixes the characteristics of the channels, Figure 1A (c); the size of the second hidden layers has been related to the number of the electrodes; the first hidden layer is only approximatively sized, as the sparsification induced by the cost function automatically find a sub-optimal size;
(7) Classification step: a softmax layer is trained by supervised learning (backprop) giving the relative probabilities of the two classes, Figure 1A (d).

The use of average operator on the time-frequency map prevents the possibility of taking advantage of the local frequencies’ distribution; nevertheless, the extracted statistical quantities yield features representing the underlying probabilistic density functions. The visual inspection of the CWT plots, computed on each channel per epoch and per subject, highlights the different periodic components in the two classes of subjects (see Figure 2). One relevant aspect of the designed deep Neural Network (NN) scheme is its ability to exploit the multichannel nature of the EEG signal, by incorporating information from all of the electrodes recordings.

### 2.5. Entropy-Based Interpretation of Hidden Layers

DL architectures aim to build and extract complex concepts from simpler ones by exploiting multilevel hierarchical structures. DL is now the leading technology to solve difficult problems formulated as artificial intelligence tasks. However, there is no general consensus on how and why DL succeeds in it. Giving a response to such delicate questions is of particular significance when such augmented intelligence is used in health problems and clinical diagnosis. The practical implementation of a DL architecture implies a feedforward neural network with its counterpart of unsolved design problems. In fact, the training of such a model on a relatively small dataset, like the ones normally available in analyzing some neurological disease, is affected by overfitting, i.e., poor performance on held-out test data. This aspect is faced here by proposing a decomposition of the available recordings in small non-overlapping parts that virtually enlarge the database size.

Another significant problem of DL approaches is related to its inability to motivate classification decisions. In this work, an attempt is made to look into the DL architecture by suggesting an information-theoretic interpretation of the latent representations at the output of the hidden layers trained by unsupervised learning, i.e., without having access to labels. The overfitting problem is structurally dealt with the concept of sparse autoencoding. The sparse autoencoder exploits, during training, the regularized cost function and a sparsity regularizer that enforces a constraint on the sparsity of the output vector generated by the hidden layers. This is obtained by constraining the hidden neurons to activate just for a limited number of training samples [20]. This effect is controlled by a coefficient of sparsity, similar to the regularization coefficient. As a result, the different training patterns, belonging to different classes, have a specialized sparse representation. In this way, the hidden output vector exhibits different probabilistic characteristics for different classes. The different classes’ representations ultimately present a different information content. In our case, the information content of such vectors is measured by means of the entropy of the distribution. The entropies of the original time signals and of the related epochs normally do not differ significantly.

## 3. Results

### 3.1. Electroencephalography (EEG) Data Preprocessing

The available EEG database described in Section 2.1 includes 10 control subjects (CNT) and six PNES patients: as previously noted this is quite a limitation being the number of subjects too small for training a DL model. Indeed, there will be many different potential settings of the weights that can model the training database almost perfectly, and each of the resulting trained networks will perform differently on the held-out test dataset, generally with lower performance than that one obtained on the training dataset. This is because the features defined within the training phase are tuned to perform well on the training dataset. The sparsity approach described in Section 2.5 can somehow reduce the impact of overfitting on the performance achieved over testing data, by preventing co-adaptation of the hidden nodes [21]; however, the number of free parameters of the network is still too high. To cope with this apparently unsolvable problem, a novel strategy has been designed. Specifically, from the EEG of each subject, 20 non-overlapping time-windows (epochs) of 5 s were extracted (by excluding parts with residual significant artefactual activity). The training dataset includes each epoch as a different record; as the database consists of 6 PNES and 10 CNT, this will yield a total cardinality of [(20 × 10) + (20 × 6)] = 320 records. The training of the two autoencoders is carried out separately and the technique of leave-one-out is used for testing (for each subject, every epoch is excluded in turn from the training phase and it is then used for testing). The same strategy was used for the classification neural network. Although the results reported in the next paragraph were achieved in this way (i.e., per epoch), the final decision on the estimated class of a subject should be taken by considering, cumulatively, the responses of the network to all of the 20 epochs of that subject. The whole DL model also includes, as a first stage, the TFM extraction: this is carried out per epoch and per channel. Figure 2 shows the average TFMs of the two classes, PNES and CNT, obtained by averaging the TFMs over the subjects, over the epochs, and over the channels. Some differences on the timing and the relative strength of the periodic components are rather evident even in the averaged maps.

### 3.2. Performance of the Deep Learning (DL) Classification System

The performance of the proposed DL architecture was quantified through standard metrics: sensitivity, specificity, positive predictive value (PPV), negative predictive value (NPV) and accuracy [22], which are defined as follows:

(3) $$\mathrm{Sensitivity}=\frac{\mathrm{TP}}{\mathrm{TP}+\mathrm{FN}}\times 100$$

(4) $$\mathrm{Specifcity}=\frac{\mathrm{TN}}{\mathrm{TN}+\mathrm{FP}}\times 100$$

(5) $$\mathrm{PPV}=\frac{\mathrm{TP}}{\mathrm{TP}+\mathrm{FP}}\times 100$$

(6) $$\mathrm{NPN}=\frac{\mathrm{TN}}{\mathrm{TN}+\mathrm{FN}}\times 100$$

(7) $$\mathrm{Accuracy}=\frac{\mathrm{TP}+\mathrm{TN}}{\mathrm{TP}+\mathrm{FN}+\mathrm{TN}+\mathrm{FP}}\times 100$$

where true positives (TP) and true negatives (TN) indicate the number of test samples correctly classified as PNES subjects or CNT; whereas, false positives (FP) and false negatives (FN) indicate the number of test examples which are wrongly detected as subjects with disease and no disease, respectively. The validity of the model was evaluated by using the standard leave-one-out procedure. It consists in excluding one record at time and training the network on the remaining records. Therefore, at each iteration, the left-out records represent the test set. The results of each testing are then averaged in order to assess the overall performance of the network.

In this study, as explained in Section 3.1, 20 EEG epochs were selected for every subject, each epoch is excluded in turn from the training phase and it is then used for testing. In this way, we ended up with 20 leave-one-out testing sessions per subject. The proposed architecture was compared with a standard shallow architecture (Support Vector Machine, SVM) (with linear and quadratic kernel) [23] and with Discriminant Analysis (with linear and quadratic discriminant function) [24]. Table 1 shows the performance of each classifier: with regard to discriminant analysis, Linear Discriminant Analysis (LDA) outperformed Quadratic Discriminant Analysis (QDA) in terms of specificity (72.1%), PPV (83.6%), NPV (73.5%) and accuracy (79.7%); whereas, SVM with linear kernel (L-SVM) provided better performance than SVM with quadratic kernel (Q-SVM), reaching a specificity of 82.5%, a PPV of 88.7%, NPV 86.5% and an accuracy of 84.4%. The DL Stacked Auto Encoders (SAE) classifier performs quite better than the other models apart from sensitivity, where Q-SVM is superior at the expenses of specificity, and PPV, where L-SVM is superior.

In Figure 3, the details of the classification performance of the proposed architecture are reported. Each bin of the representation refers to the output of the softmax layer obtained on each one of the 20 leave-one-out testing epochs; each sub-plot illustrates the results of the 20 leave-one-out testing sessions of the single subject. The height of each bin is the estimated output of the classification network obtained in the corresponding leave-one-out testing session (one correct classification; 0 misclassification). The two categories (PNES and CNT) are shown separately. The red dotted line is the mean output level of the network, averaged over the 20 testing sessions. The accuracy of the classification achieved by considering cumulatively the epochs referring to a single subject is 100%. To further validate the model, a fresh EEG from an additional PNES subject has been processed through the DL chain. 20 epochs of 5 s time signal have been extracted from the EEG recording and they have been correctly classified in 17 over 20 blocks, corresponding to a 85% accuracy.

A sensitivity analysis has been carried out in order to note features from what electrodes (brain areas) are mainly relevant for classification. This analysis uses the weights’ matrices of the trained DL network. According to some clinical results in the PNES literature, some electrodes of the frontal and occipital areas appear to be most informative [25].

### 3.3. Entropic Interpretation of DL Classification

**Figure 4:**
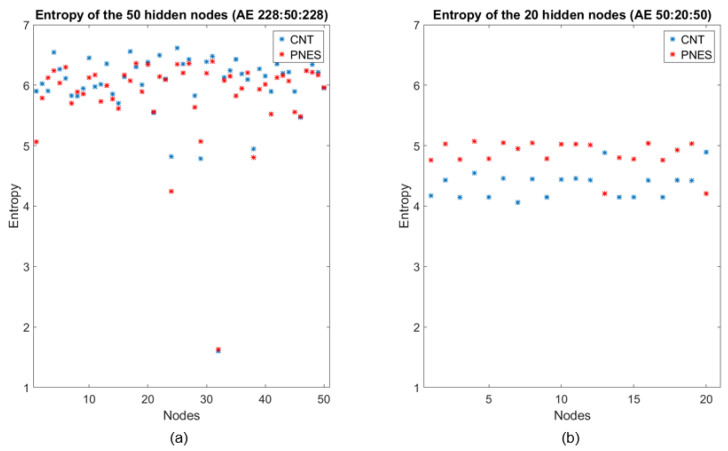
Entropy representation of PNES (red dots) and CNT (blue dots) evaluated at the outputs of the hidden nodes of the two compressed representations. (**a**) Entropy values related to PNES and CNT features extracted from the first AE (50 × 1). At this stage, the entropies of the two classes are comparable; (**b**) Entropy values related to PNES and CNT features extracted from the second AE (20 × 1). At this stage, the entropies decrease and they are different for the two classes and generally greater for PNES than CNT.

To interpret the behavior of DL and thus trying to “opening the black box”, an analysis of the output of the two hidden layers (the first with 50 nodes, the second with 20 nodes), in the two stages of compression through encoding, was carried out. Specifically, a set of 50 (or 20) vectors was obtained by considering the output of each hidden node of the first (or the second) compressed representation corresponding to all of the available epochs. Then, the Shannon entropy (SE) of such node vectors was estimated. Figure 4 reports the SE values of the hidden layers’ nodes of the two successive autoencoders. The results suggest three interesting comments:

(1) As recently noted in the literature [21], most of the information encoded in the input epochs is exploited in compression to generate an efficient representation regardless of the training labels, as the compression phase ignores the labels (considered just in the final classification stage);
(2) The mean entropy indeed decreased as the layers deepened, which is intuitively rather expected as the successive representations gradually build the final vectors’ representation [26];
(3) In contrast to the first stage of compression, the hidden layer of the second encoder seems clearly extracting the class information, i.e., the latent differences between the classes, even though in absence of any label information. This is an original result not previously reported in the literature, at our best knowledge. This is the first study where the behavior of the compressing stages has been discussed from an information-theoretical perspective in classification networks. In our opinion, the noted behavior can justify the use of a deep structure to extract high-level features that can widely facilitate the classification procedure [27].

A question may arise, if the entropies of the original time signals can already highlight the difference between the two classes. An analysis has been carried out by computing both Shannon Entropy and normalized Permutation Entropy on the EEG signals (see Supplementary Materials, Figure S1), by taking the global average on subjects, epochs and electrodes: the result is that there are no significant statistical differences on the entropies computed on the two classes.

## 4. Discussion and Conclusions

The recent emergence of DL methods in many diverse application domains has motivated such a data-driven approach in difficult clinical diagnosis problems. In this work, a deep architecture was proposed to help the discrimination of PNES subjects from healthy controls through routinely EEG. The proposed approach can be useful for the early identification of such patients. The proposed deep architectural scheme includes a first level, where time-frequency features are extracted by CWT, two compressing stages implemented by autoencoders (SAE), and a final classification network with softmax nonlinearity. The first stage does not require learning; SAE are trained off-line by unsupervised learning, and the classification network is trained by backpropagation. A final supervised fine-tuning step can be applied to the whole structure. DL-based systems are claimed to be able to extract higher level features directly from the available data, in such a way also reducing noise and rejecting non relevant information. One of the problems of DL is the difficulty of explaining its behavior and achievements, which is a strong limitation in clinical settings. A second limitation, particularly pertinent to EEG-based clinical applications, is the availability of limited data. In this work, the two problems are faced as follows. As regards the difficult interpretation of DL results, a guided transformation of the data, through the estimation of the time-frequency maps (TFM), was carried out (at this stage, EEG channels are treated separately). Then, the information extracted from the TFMs of the channels are combined through a double-level SAE. The limited EEG data were virtually augmented by segmenting each EEG recording into 20 non-overlapping epochs; which is a good strategy also to cope with the different length of the recordings and the presence of residual artifacts. The use of both a regularizer and a sparsity constraint allowed to reduce the impact of overfitting due to the limited size of the dataset. As an alternative to sparsity, the random dropout of the hidden nodes was previously proposed in the literature [28]. Finally, the present paper proposed an information-theoretic approach to investigate the behavior and the performance of the deep model; specifically, estimating the SE of the output of the second AE level unveiled that DL is able to autonomously extract the class information without the need of labels. This evidently facilitates the subsequent classification stage and may explain the power of DL schemes. The performance of the DL model resulted good (86% accuracy) and better than some standard shallow approaches.

The main limitations of this work is clearly the number of available subjects. From an architectural design perspective, a more detailed sensitivity study should be carried out by considering different sizes and levels of the AEs. A more detailed analysis of the TFMs could be of great help to generate more appropriate features than the ones here proposed. Finally, a visual representation of the features extracted by the DL chain at various levels could be advantageous in order to associate the features to the brain areas of the original signals. Here, we just analyzed the trained weights’ matrices in order to qualitatively assess the relative importance of the electrodes.

## Figures and Tables

**Table 1:** Performance of the proposed system compared to other classification systems.

Classifier	Sensitivity (%)	Specificity (%)	PPV (%)	NPV (%)	Accuracy (%)
SAE	88.8	90.7	86.2	88.6	86.5
LDA	84.1	72.1	83.6	73.5	79.7
QDA	88.0	54.2	76.2	73.3	75.3
L-SVM	88.0	82.5	88.7	86.5	84.4
Q-SVM	92.3	57.5	78.6	85.2	80.3

## Supplementary Materials

The following are available online at http://www.mdpi.com/1099-4300/20/2/43/s1, Figure S1: Permutation Entropy Boxplots of the healthy control (CNT) and the psychogenic non-epileptic seizures (PNES).
