# Supplementary figures and images for: Information Theoretic-Based Interpretation of a Deep Neural Network Approach in Diagnosing Psychogenic Non-Epileptic Seizures
Source: Entropy (Basel). 2018 Jan 23;20(2):43. doi: 10.3390/e20020043 (PMC7512641; doi:10.3390/e20020043)

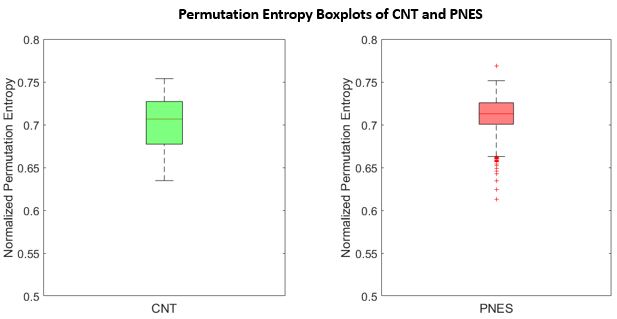

Supplement: Supplementary file 1 [file entropy-20-00043-s001.zip › entropy-251951-supplementary.JPG]
